# What Are the Burden, Causes, and Costs of Early Hospital Readmissions After Kidney Transplantation?

**DOI:** 10.1177/15269248211003563

**Published:** 2021-03-24

**Authors:** Olusegun Famure, Esther D. Kim, Magdalene Au, Roman E. Zyla, Johnny W. Huang, Pei Xuan Chen, Yanhong Li, S. Joseph Kim

**Affiliations:** 1 Division of Nephrology and the Kidney Transplant Program, Toronto General Hospital, University Health Network, University of Toronto, Toronto, Ontario, Canada; 2 Department of Medicine, University of Toronto, Toronto, Ontario, Canada; 3 Institute of Health Policy, Management and Evaluation, University of Toronto, Toronto, Ontario, Canada

**Keywords:** readmission, kidney transplantation, incidence, risk factors

## Abstract

**Introduction:**

Kidney transplant recipients are at risk for complications resulting in early hospital readmission. This study sought to determine the incidences, risk factors, causes, and financial costs of early readmissions.

**Design:**

This single-centre cohort study included 1461 kidney recipients from 1 Jul 2004 to 31 Dec 2012, with at least 1-year follow-up. Early readmission was defined as hospitalization within 30 or 90-days postdischarge from transplant admission. Associations between various parameters and 30 and 90-days posttransplant were determined using multivariable Cox proportional hazards models. The hospital-associated costs of were assessed.

**Results:**

The rates of early readmission were 19.4% at 30 days and 26.8% at 90 days posttransplant. Mean cost per 30-day readmission was 11 606 CAD. Infectious complications were the most common reasons and resulted in the greatest cost burden. Factors associated with 30 and 90-days in multivariable models were recipient history of chronic lung disease (hazard ratio or HR 1.78 [95%CI: 1.14, 2.76] and HR 1.68 [1.14, 2.48], respectively), median time on dialysis (HR 1.07 [95% CI: 1.01, 1.13]and HR 1.06 [95% CI: 1.01, 1.11], respectively), being transplanted preemptively (HR 1.75 [95% CI: 1.07, 2.88] and HR 1.66 [95% CI: 1.07, 2.57], respectively), and having a transplant hospitalization lasting of and more than 11 days (HR 1.52 [95% CI: 1.01, 2.27] and HR 1.65 [95% CI: 1.16, 2.34], respectively).

**Discussion:**

Early hospital readmission after transplantation was common and costly. Strategies to reduce the burden of early hospital readmissions are needed for all patients.

## Introduction

Kidney transplantation is the treatment of choice for the majority of patients with end-stage kidney disease.^1^ However, during the immediate posttransplant period, patients are at an elevated risk for a number of potentially serious medical or surgical complications. In many cases, these complications result in patients being readmitted to hospital. Previous studies have identified a hospitalization rate of greater than 30% within the first 30-days after discharge from the initial transplant hospitalization.^2,3^ Not only does this reflect a reduction in a patient’s health status, it also represents a significant burden on healthcare delivery systems. In the United States, early hospital readmissions after kidney transplantation result in costs of approximately 10 000 USD for each episode.^3^ Developing an understanding of the risk factors and causes of early hospital readmissions is essential to identify strategies for reducing their burden on both patients and hospitals.

Risk factors for readmission post-kidney transplant fall into two broad categories. The first represents characteristics reflective of a reduced overall health status that are often associated with increased morbidity and mortality in the general population, including recipient age or frailty.^3,4^ The other primary category includes those risk factors which are directly associated with kidney transplantation, including donor age and expanded criteria donor (ECD) transplants.^3^ The principal causes of hospital readmissions across different studies vary due to differences in categorization and time posttransplant.^5^ In the early posttransplant period, surgical complications and rejection have been shown to be particularly significant reasons for readmission.^2^

While early hospital readmissions have been systematically studied in the United States, differences in healthcare delivery and transplant outcomes may not allow extrapolation from these studies to other healthcare systems. Studies of early hospital readmissions in kidney transplant recipients have been less common in Canadian transplant populations.^6^ Recently, we undertook a population-based study of Ontario kidney transplant recipients and examined secular trends in the incidence of 30-day readmissions after transplantation.^7^ This analysis showed a stable rate of 21% from 2002 to 2014. However, a detailed evaluation of causes and costs of readmission was not undertaken.

## Specific Aims

Considering the gaps in knowledge about the epidemiology of early hospital readmissions in a Canadian kidney transplant population, we conducted a study examining the incidence, risk factors, causes, and costs of hospitalizations in the first 30-days post-discharge after kidney transplantation in our large, single-center Canadian cohort. To capture a larger spectrum of patients at-risk for hospital readmission, we also extended the period of risk for readmissions to 90-days after discharge from the transplant admission.

## Methods

### Design and Setting

This is an observational cohort study using the Comprehensive Renal Transplant Information System at the Toronto General Hospital, University Health Network.^8^ The research ethics board at the University Health Network approved this study.

### Population

From July 1, 2004 to December 31, 2012, a total of 1,165 kidney transplants were performed, with 584 (50.1%) from living donors. Mean age of the cohort was 48.8 years, 37.7% were female, 69.7% were White, and the cause of end-stage kidney disease were predominantly from glomerulonephritis (33.2%) and diabetes mellitus (26.8%).

### Sampling of the Population

All adult (age ≥ 18 years) kidney transplant recipients receiving a transplant from July 1, 2004 to December 31, 2012 (followed until December 31, 2013) at the Toronto General Hospital were eligible for study inclusion. Exclusion criteria included: (*a*) multi-organ transplant recipients, (*b*) simultaneous organ transplant recipients, (*c*) transplants from outside institutions, (*d*) primary non-function, and (*e*) death, graft failure, or lost-to-follow up prior to discharge from the transplant hospitalization. The latter was included as an exclusion criterion since patient how die or lose their graft prior to discharge from their transplant hospital stay cannot be at risk for early hospital readmission after transplantation.

### Variables Definition and Data Collection

In the primary analysis, early hospital readmission after transplant was defined as the first re-admission occurring between 1 and 30 days after discharge from the transplant hospitalization. First readmissions occurring between 1 and 90 days after discharge from the transplant hospitalization were examined in a secondary analysis. Causes of early hospital readmission were identified using manual chart review of the diagnoses provided in discharge summaries available in the electronic health record system, along with those summaries retrieved from outside hospitals where patients may have been admitted. Baseline recipient, donor, and transplant characteristics were considered as potential risk factors for early readmission and were collected at discharge from the transplant hospitalization. Recipient characteristics included age, sex, race, cause of end-stage kidney disease, history of diabetes mellitus, history of chronic lung disease, body mass index at discharge, time on dialysis, and kidney function at baseline. Kidney function was measured using estimated glomerular filtration rate (eGFR), which was calculated using the CKD-EPI equation.^9^ Donor characteristics included age, history of hypertension, and donor type (living versus deceased). Transplant factors included length of transplant hospitalization, delayed graft function, acute rejection during the transplant hospitalization, type of induction therapy (interleukin-2 receptor blocker vs. rabbit anti-thymocyte globulin), type of calcineurin inhibitor at discharge (cyclosporine vs. tacrolimus), and transplant era.

Inpatient cost data was obtained from the University Health Network Accounting Centre and adjusted for inflation to the value of 2013 Canadian dollars using the Consumer Price Index, health and personal care.^10^ The costs of inpatient care were captured across various domains including laboratory testing, medical imaging, pharmacy, other treatments (eg, dialysis), and facility-related expenditures (eg, room and board). Cost information was assessed solely from a hospital perspective; no community-based cost data was included in this study.

### Data Analysis

A descriptive analysis was performed for all study variables and all distributions were examined. Categorical data were described using frequencies/percentages and differences across groups were examined using the chi-square test. Continuous variables were described using the mean (standard deviation) for normally distributed data and differences across groups were assessed using the Student t-test. The median (interquartile range) was used to describe non-normally distributed data and differences across groups were compared using the Wilcoxon rank sum.

To investigate the association of recipient, donor, and transplant characteristics with early hospital readmission, multiple analyses were performed. The Kaplan-Meier product limit method was used to assess time to first early readmission. The Cox proportional hazards model was used to estimate the independent association between recipient, donor, and transplant characteristics with first early readmission, after adjustment for baseline covariates. The method of Multiple Imputation by Chained Equations was used to address missing covariate data. A two-tailed *P* value of < 0.05 was considered statistically significant. All analyses were performed using Stata/MP 12.0 (StataCorp, College Station, TX).

## Results

**Figure 1.**
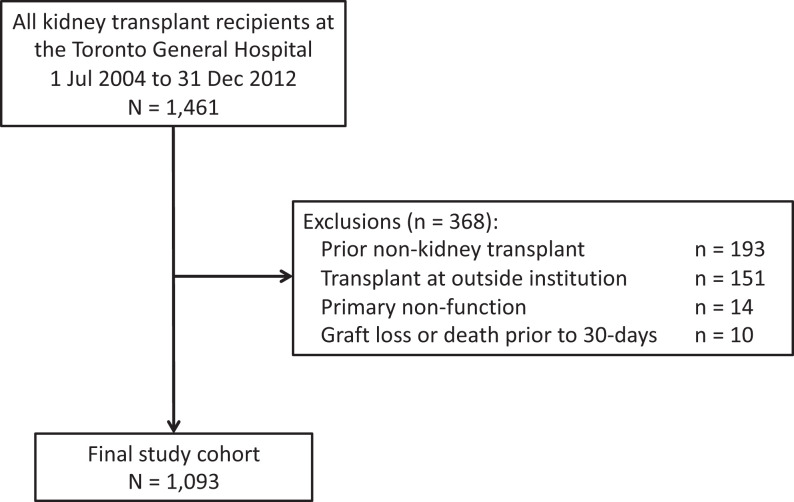
Study flow diagram.

**Figure 2.**
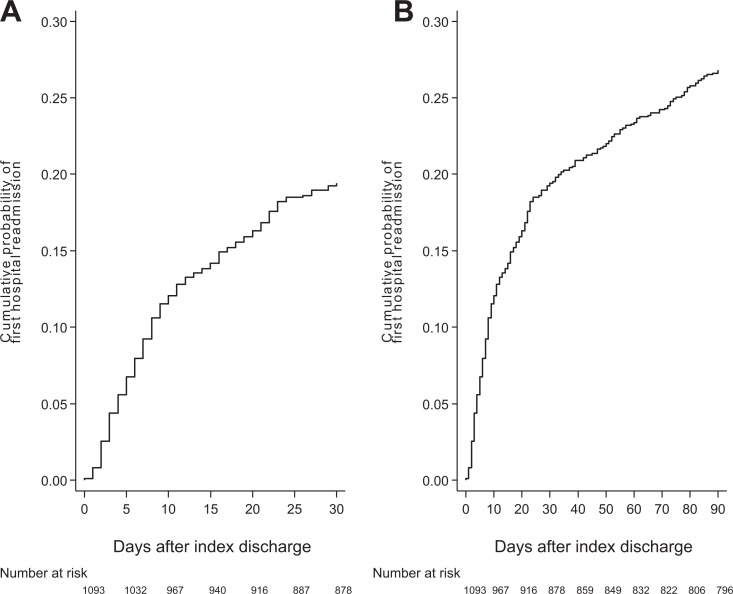
Cumulative probability of early hospital readmission within 30-days (A) and 90-days (B) following discharge from transplant hospitalization.

We identified 1461 patients who were transplanted between July 1, 2004 and December 31, 2012 at the Toronto General Hospital, University Health Network. **Figure 1** shows the results of applying the exclusion criteria to obtain the final cohort of 1093 patients. Within this cohort, the mean age was 49.9 years, 38.9% were female, and 65.4% were White (**Table 1**). The cumulative probability of early readmission at 30- and 90- days posttransplant were 19.4%, and 26.8%, respectively (**Figure 2**). Median time to first readmission within 30-days was 8 days (IQR 4, 16) and within 90-days was 13 days (IQR 5, 35) (**Table 2**). Median duration of within 30 and 90 days were both 5 days (IQR 2, 9). Mean hospital cost per 30-day was 11 606 CAD (SD 15113; median 7023.6; IQR 3860.8, 12,404). Departments with high mean costs included laboratory, pharmacy, room and board, medical imaging, and cardiology services (Supplementary Table 1).

**Table 1.** Recipient, Donor, and Transplant Characteristics.

Variables	Number of patients (N = 1093)	Characteristic
Recipient characteristics	N	
Mean recipient age at transplant (± SD), years	1093	49.9 (13.2)
Recipient male sex	668	61.1%
Recipient non-white race	375	34.6%
Smoker	467	43.4%
History of diabetes mellitus	275	25.2%
History of cardiovascular disease	269	24.6%
History of chronic lung disease	71	6.5%
Mean recipient body mass index at discharge (SD), kg/m2	1,072	26.7 (5.8)
Mean recipient eGFR at baseline (SD), ml/min	1,083	60.6 (26.3)
Median time on dialysis [IQR], years	1,093	3.4 [1.2, 5.9]
Peak PRA > 0%	560	51.4%
Dialysis modality at the time of transplant		
Conventional hemodialysis	612	56.3%
Home hemodialysis	107	9.8%
Peritoneal dialysis	239	22.0%
Pre-emptive	129	11.9%
Donor characteristics		
Mean donor age at transplant (SD), years	1,086	46.3 (14.2)
Median donor body mass index (SD), kg/m2	1,077	26.7 (5.3)
Deceased donors	543	49.7%
Expanded criteria donor	169	15.5%
Donation after circulatory death	99	9.1%
Transplant characteristics		
Delayed graft function	212	19.4%
Biopsy-proven acute rejection	45	4.1%
Depleting induction therapy (vs. non-depleting)	797	72.9%
Tacrolimus at the time of discharge (vs. cyclosporine)	917	85.5%
Transplant era		
2004-2007	386	35.3%
2008-2010	418	38.2%
2011-2012	289	26.4%

**Table 2.** Incidence and Duration of Early Hospital Readmissions.

Outcomes	Within 30 days	Within 90 days
All early readmissions	229	392
Median duration of all early readmissions	5 (2, 9) days	5, (2, 9) days
First early admission	212	292
Incidence rate for first early readmission	22.60 (95% CI: 19.76, 25.86) per 100 person-months	11.31 (95% CI: 10.09, 12.69) per 100 person-months
Median time to first early readmission	8 (4, 16) days	13 (5, 35) days

Infectious complications, kidney/genitourinary factors, and rejection were the most likely reasons for readmission within both 30 and 90-day readmission (Tables 3). Within 30-days posttransplant, 21.0% of readmission events were attributable to infections and within that 46% of infectious readmissions were because of urinary tract infections (Supplementary Table 2a). A higher proportion of readmissions due to infections (28.1%) was observed within 90-days posttransplant compared to within 30-days (Table 3). The proportion of patients readmitted with rejection was similar between 30 and 90-days posttransplant (20.5% vs. 18.1%, respectively) (Table 3). For both groups, T-cell mediated rejection was the leading cause of rejection-related readmissions (58.8% and 58.0%, respectively) (Supplementary Tables 2b). Except for infection, all other reasons of readmissions including kidney/genitourinary, surgical complications, drug toxicity, cardiovascular, gastrointestinal, and endocrine reasons, appeared in higher proportion within 90-days compared to 30-days (Supplementary Tables 3a and 3b). The reasons for 30-day readmissions with the highest mean cost were surgical complications ($20,714 CAD [SD 29,145]), rejection ($19,618 CAD [SD 18,832]), and infection ($11,894 CAD [SD 17,373]) (Table 3). Considering the frequency of readmissions within 30-days, the greatest cost burden was attributable to rejection, infection, and kidney/genitourinary issues.

**Table 3.** Reasons for Hospital Readmission Within 30- and 90-Days and Mean Costs Per Early Hospital Readmission.

Primary reasons	30-Day Cases N (%)	Mean cost (CAD$) per early hospital readmission (SD)	90-Day Cases N (%)
Infection	48 (21.0)	11 894 (17 373)	110 (28.1)
Renal and genitourinary	47 (20.5)	10 271 (10 338)	71 (18.1)
Rejection	34 (14.9)	19 618 (18 831)	50 (12.7)
Drug toxicity	19 (8.3)	6 170 (4902)	26 (6.6)
Surgical complication	17 (7.4)	20 714 (29 145)	25 (6.4)
Cardiovascular	12 (5.2)	6794 (2894)	20 (5.1)
Gastrointestinal	4 (1.8)	5801 (1437)	11 (2.8)
Endocrine	2 (0.9)	8812 (2400)	4 (1.0)
Other	42 (18.3)	7734 (7573)	68 (15.4)
Missing	4 (1.8)	8403 (10 186)	7 (1.8)
Total	229	11 606 (15 113)	392

Univariable models showed that factors associated with an increased risk of readmissions within both 30 and 90-days posttransplant was recipient history of chronic lung disease (Supplementary Table 4). Receiving non-depleting agent as type of induction therapy at transplant date was associated with decreased risks of readmission within 30-days posttransplant. Being transplanted in more recent years was associated with increased risks of readmission within 30-days posttransplant only. Length of transplant hospitalization of and more than 11 days was associated with increased risks of readmission within 90-days posttransplant. Additionally, median time on dialysis was only associated with the 90-days readmission model.

In the adjusted multivariable models (Table 4), recipient history of chronic lung disease, median time on dialysis, being transplanted pre-emptively rather than having had conventional hemodialysis, and having a transplant hospitalization lasting of and more than 11 days were associated with elevated risks of readmission within both 30 and 90-days posttransplant (HR 1.87 [95% CI: 1.20, 2.92], HR 1.07 [95% CI: 1.01, 1.13], HR 1.75 [95% CI: 1.07, 2.88], and HR 1.52 [95% CI: 1.01, 2.27] respectively for 30-days readmission; HR 1.78 [95% CI: 1.21, 2.62], HR 1.06 [95% CI: 1.01, 1.11], HR 1.66 [95% CI: 1.07, 2.57], and HR 1.65 [95% CI: 1.16, 2.34] respectively for 90-days readmission). When the causes for readmission were evaluated across pre-emptive and non-pre-emptive patients (the latter stratified by deceased or living donors), there were no systematic differences notable across groups (Supplementary Table 5). Meanwhile, having non-depleting agent as the type of induction therapy at transplant was associated with decreased risk of 30-day readmission (HR 0.65 [95% CI: 0.44, 0.96]) and having elevated peak PRA of more than 0% was associated with decreased risk of 90-day readmission only (HR 0.76 [95% CI: 0.59, 0.99]).

**Table 4.** Association of Risk Factors with Early Hospital Readmission by Multivariable Cox Proportional Hazards Model.

Variables	Early hospital readmission within 30-days		Early hospital readmission within 90-days	
	HR (95% CI)	P value	HR (95% CI)	P value
Recipient age at transplant, years	1.00 (0.98, 1.01)	0.42	1.00 (0.99, 1.01)	0.42
Recipient sex				
Male	*ref*		*ref*	
Female	0.89 (0.67, 1.20)	0.45	0.98 (0.77, 1.26)	0.89
Recipient race				
White	*ref*		*ref*	
Non-White	0.91 (0.66, 1.25)	0.55	0.99 (0.76, 1.29)	0.93
Recipient history of diabetes mellitus	1.31 (0.94, 1.85)	0.12	1.28 (0.96, 1.71)	0.10
Recipient history of chronic lung disease	1.87 (1.20, 2.92)	0.01	1.78 (1.21, 2.62)	0.004
Recipient history of cardiovascular disease	0.88 (0.62, 1.26)	0.49	0.98 (0.73, 1.32)	0.90
Recipient body mass index at discharge, kg/m2	1.03 (1.00, 1.05)	0.05	1.02 (1.00, 1.04)	0.09
Recipient eGFR at baseline, ml/min	1.00 (1.00, 1.01)	0.29	1.00 (1.00, 1.01)	0.49
Peak PRA, %				
0%	*ref*		*ref*	
>0%	0.80 (0.59, 1.08)	0.14	0.76 (0.59, 0.99)	0.04
Time on dialysis, years	1.07 (1.01, 1.13)	0.02	1.06 (1.01, 1.11)	0.02
Dialysis modality at the time of transplant				
Conventional hemodialysis	*ref*		*ref*	
Home hemodialysis	1.36 (0.87, 2.14)	0.18	1.32 (0.89, 1.95)	0.16
Peritoneal dialysis	1.14 (0.80, 1.63)	0.47	1.10 (0.81, 1.49)	0.55
Pre-emptive	1.75 (1.07, 2.88)	0.03	1.66 (1.07, 2.57)	0.02
Donor age at transplant, years	1.01 (0.99, 1.02)	0.41	1.01 (1.00, 1.01)	0.29
Type of donation				
Deceased	*ref*		*ref*	
Living	1.11 (0.73, 1.70)	0.62	1.02 (0.72, 1.47)	0.82
Expanded criteria donor (ECD)				
Non-ECD	*ref*		*ref*	
ECD	1.01 (0.60, 1.71)	0.97	0.95 (0.61, 1.47)	0.82
Donation after circulatory death	1.02 (0.61, 1.71)	0.94	0.94 (0.61, 1.46)	0.80
Length of transplant hospitalization, days				
≤7 days	*ref*		*ref*	
8-10 days	1.13 (0.78, 1.62)	0.52	1.31 (0.95, 1.79)	0.10
>11 days	1.52 (1.01, 2.27)	0.04	1.65 (1.16, 2.34)	0.01
Delayed graft function	0.89 (0.59, 1.33)	0.56	0.97 (0.70, 1.36)	0.88
Biopsy-proven acute rejection	0.81 (0.39, 1.66)	0.56	0.94 (0.53, 1.69)	0.85
Induction therapy at transplant date				
Depleting agent	*ref*		*ref*	
Non-depleting agent	0.65 (0.44, 0.96)	0.03	0.79 (0.57, 1.09)	0.15
Types of calcineurin inhibitor at discharge				
Tacrolimus	*ref*		*ref*	
Cyclosporine	1.07 (0.71, 1.61)	0.74	1.10 (0.78, 1.55)	0.60
Transplant era				
2004 - 2007	*ref*		*ref*	
2008 - 2010	1.18 (0.82, 1.70)	0.36	1.28 (0.94, 1.72)	0.11
2011 - 2012	1.25 (0.85, 1.86)	0.26	1.22 (0.87, 1.71)	0.25

Abbreviations: eGFR, estimated glomerular filtration rate; PRA, panel reactive antibody.

## Discussion

Using a large, single-center cohort of kidney transplant recipients, we identified the incidence, causes, risk factors, and costs of early posttransplant readmissions in a Canadian kidney transplant population. Hospital readmissions within 30- and 90-days posttransplant were common and costly. The most frequent reasons for readmission within 30 and 90-days were infection, rejection, and kidney/genitourinary issues. Through multivariable analysis, we identified recipient history of chronic lung disease, time on dialysis before transplant, being pre-emptively transplanted and prolonged transplant hospitalization as independent risk factors for both 30 and 90-day readmissions. Posttransplant readmissions result in both clinical and financial burdens, suggesting a need for quality improvement initiatives to identify and prevent avoidable readmissions.

Although kidney transplantation is the preferred treatment for end-stage kidney disease, recipients are at increased risk for surgical and medical complications during the peri- and posttransplant period. Previously, early hospital readmission rates have been examined in kidney transplant populations as a surrogate measure of posttransplant complications and care processes. Studies in the United States have shown substantial variability in 30-day readmissions rates between transplant centers. While McAdams-Demarco et al. reported that 30.5% of recipients experience a 30-day readmission, rates ranging from 11% to 47% have been reported for single-centers.^2,3,11,12^ The 30-day readmission rate of 19.4% measured in this study was comparable to the rate of 20.8% across all Ontario kidney transplant centers and compares favorably to US experience.^7^ The geographic disparities in early hospital readmission rates may be attributed to differences in patient populations as well as center- and system-level practices.^6^ Multinational studies of readmission rates for different medical and surgical hospitalizations have found variability across countries.^13^ Along with the clinical burden of posttransplant readmissions, we also characterized the financial and resource burden. The mean cost per 30-day readmission calculated in this study was comparable to those reported by Englesbe et al. ($9962 USD) and McAdams-Demarco et al. ($10 551 USD).^3,14^

Infections were the most frequent reason for both 30- and 90-day readmissions, the tendency of increased infectious events within the first 3 months after transplant has been well-documented in kidney recipients.^2,15^ While previous studies have focused on the 30-day readmission metric, we also included the 90-day readmission metric to examine temporal trends in readmissions. The causes and risk factors for readmission were similar for 30-day and 90-day readmissions, but an increased proportion of readmissions were attributable to infections over the 90-days post-discharge. This may reflect the changing risk of infection over the early and intermediate posttransplant period. Infections within the first 30-days are more likely to be attributed to surgical complications or donor and recipient-derived pathogens while infections in the subsequent 5 months are more likely to be opportunistic infections due to immunosuppression.^16,17^ Consequently, measures of 30-day readmissions in kidney transplant populations may underestimate the burden of infectious complications necessitating readmission. This may merit further study given that infection was the most common reason for readmission and resulted in the greatest cost burden.

In our study sample, urinary tract infections were the most common type of infection that contributes to both 30- and 90-day readmissions, which was consistent with literature findings.^18^ Use of immunosuppressive drugs, exposure to nosocomial pathogens and need for invasive urinary and intravascular devices put recipients at greater risk of developing urinary infection-related readmissions.^18^ Our center currently does not have any prophylactic antibiotic protocols since strong evidence for their use is lacking. In addition, careful considerations of the interaction between antibiotic medication and immunosuppression and infection with resistant bacteria are always imperative when deciding on a prophylactic measure.^19^

Infection, rejection, and kidney/genitourinary issues have also been found to be common reasons for readmission in several other studies of posttransplant early hospital readmissions.^2,3,11,20^ Direct comparisons across different studies are difficult due to differences in coding of reasons for readmission as well as variations in the inclusion of planned versus unplanned readmissions. Notably, readmissions for surgical complications were less frequent in the current cohort compared to other studies.^2,11,20^

Previous studies have sought to identify patient, transplant, and center-level risk factors for posttransplant early hospital readmissions. While there has been substantial variability in the specific risk factors identified in each study, factors related to recipient comorbidity.^3,4,11,20^ and poor health literacy,^11^ marginal donor kidneys,^3^ care processes (eg weekend discharge)^2^ and in-hospital adverse events^2,11^ have been associated with increased risk of early readmission. The identification of a history of chronic lung disease, longer time on dialysis before transplant and prolonged transplant hospitalization as risk factors for early readmissions in this cohort suggest that patient comorbidity and in-hospital adverse events were important factors leading to readmission. Chronic lung disease was also reported by McAdams-Demarco et al. as a risk factor for 30-day readmissions.^3^ Longer exposure to dialysis^21^ and longer length of transplant stay^3^ are both intuitive and previously reported risk factors associated with early readmissions. Dialysis duration has been suggested as the strongest independent modifiable risk factor for kidney transplant recipients because of its association with increased comorbidity burden, immunological alterations, and physiological reserve decline in recipients.^5,22^ Meanwhile, longer hospital stay after transplant has been attributed to the disease status of the recipient and the organ.^6^ Prolonged transplant admission, therefore, is likely a reflection of a high-risk recipient who is prone to adverse outcomes such as early readmissions. Early identification of such patients may be beneficial in assisting to develop tailored quality-improvement interventions similar to the program reported by Taber et al. for patients with delayed graft function.^23^ Other in-hospital complications that have been reported as risk factors for early readmission include delayed graft function and electrolyte abnormalities.^20^ Since in-hospital complications are generally factors that are quite visible at discharge, they may be risk factors that are readily identifiable but difficult to modify.

A notable and unexpected risk factor found to be associated with both 30- and 90-day readmission was being pre-emptively transplanted. Pre-emptive transplantation has been shown to be associated with better posttransplant outcomes because recipients are generally healthier. However, factors that may explain a higher likelihood for readmission include the inability to maximize the use of native kidney function, the failure to take advantage of putative immunosuppressive effects of uremia, and the potential for reduced adherence to immunosuppressive medications due to the lack of experience of the morbidity of dialysis.^24,25^ Alternatively, this association may be spurious due to inadequate capture of factors that may relate to pre-emptive transplant status and the tendency to early hospital readmission (ie residual confounding).

Centre-level differences in care practices may also affect the risk factors for early readmission.^3,26^ For example, the risk for recipients with diabetes may be mitigated through interprofessional discharge preparation and care in the early posttransplant period, including the use of a transplant discharge coordinator, diabetes educator, and dedicated transplant pharmacy.^26^ Healthcare system-level factors, such as access to care, may also be important, but difficult to ascertain given the paucity of international studies on posttransplant early readmissions.

The present report is the first Canadian study to rigorously examine the incidence, causes, risk factors and costs of temporally relevant early hospital readmissions in a large, single-center cohort of kidney transplant recipients. Despite its strengths, our study has several limitations. Since this was a single-center study, there may be limits to its generalizability. Moreover, we were unable to assess center-level factors. Despite the extensive list of covariates that were included in the multivariable analyses, we were unable to capture other potentially important risk factors such as frailty and social support. Another important limitation was that readmissions to other hospitals among patients were not captured. However, we estimate that this would not have a substantial effect on the current results since patients are likely to be readmitted to the transplant center in the early posttransplant period. Finally, there is the potential for residual confounding from unmeasured factors, which is inherent in all observational studies.

## Conclusion

Hospital readmissions within 30 and 90-days post-discharge resulted in clinical and financial burdens for kidney transplant recipients at a large Canadian transplant center and the healthcare system at large. Further work is needed to evaluate the posttransplant outcomes associated with early readmissions, to elucidate the role of preventable causes. and to develop effective interventions to reduce the risk of readmissions in this high-risk patient population. This effort may be facilitated by the development of prediction models to identify the patients at highest risk for first and recurrent hospital readmissions. Careful evaluation of relevant predictors, and how they should be incorporated into statistical models, will be necessary to maximize predictive accuracy.^5^

## Supplemental Material

Supplemental Material, sj-docx-1-pit-10.1177_15269248211003563 - What Are the Burden, Causes, and Costs of Early Hospital Readmissions After Kidney Transplantation? Click here for additional data file. Supplemental Material, sj-docx-1-pit-10.1177_15269248211003563 for What Are the Burden, Causes, and Costs of Early Hospital Readmissions After Kidney Transplantation? by Olusegun Famure, Esther D. Kim, Magdalene Au, Roman E. Zyla, Johnny W. Huang, Pei Xuan Chen, Yanhong Li and S. Joseph Kim in Progress in Transplantation

Supplemental Material, sj-docx-2-pit-10.1177_15269248211003563 - What Are the Burden, Causes, and Costs of Early Hospital Readmissions After Kidney Transplantation? Click here for additional data file. Supplemental Material, sj-docx-2-pit-10.1177_15269248211003563 for What Are the Burden, Causes, and Costs of Early Hospital Readmissions After Kidney Transplantation? by Olusegun Famure, Esther D. Kim, Magdalene Au, Roman E. Zyla, Johnny W. Huang, Pei Xuan Chen, Yanhong Li and S. Joseph Kim in Progress in Transplantation

Supplemental Material, sj-docx-3-pit-10.1177_15269248211003563 - What Are the Burden, Causes, and Costs of Early Hospital Readmissions After Kidney Transplantation? Click here for additional data file. Supplemental Material, sj-docx-3-pit-10.1177_15269248211003563 for What Are the Burden, Causes, and Costs of Early Hospital Readmissions After Kidney Transplantation? by Olusegun Famure, Esther D. Kim, Magdalene Au, Roman E. Zyla, Johnny W. Huang, Pei Xuan Chen, Yanhong Li and S. Joseph Kim in Progress in Transplantation

Supplemental Material, sj-docx-4-pit-10.1177_15269248211003563 - What Are the Burden, Causes, and Costs of Early Hospital Readmissions After Kidney Transplantation? Click here for additional data file. Supplemental Material, sj-docx-4-pit-10.1177_15269248211003563 for What Are the Burden, Causes, and Costs of Early Hospital Readmissions After Kidney Transplantation? by Olusegun Famure, Esther D. Kim, Magdalene Au, Roman E. Zyla, Johnny W. Huang, Pei Xuan Chen, Yanhong Li and S. Joseph Kim in Progress in Transplantation

Supplemental Material, sj-docx-5-pit-10.1177_15269248211003563 - What Are the Burden, Causes, and Costs of Early Hospital Readmissions After Kidney Transplantation? Click here for additional data file. Supplemental Material, sj-docx-5-pit-10.1177_15269248211003563 for What Are the Burden, Causes, and Costs of Early Hospital Readmissions After Kidney Transplantation? by Olusegun Famure, Esther D. Kim, Magdalene Au, Roman E. Zyla, Johnny W. Huang, Pei Xuan Chen, Yanhong Li and S. Joseph Kim in Progress in Transplantation
