# Supplementary material for: What Are the Burden, Causes, and Costs of Early Hospital Readmissions After Kidney Transplantation?
Source: Prog Transplant. 2021 Mar 24;31(2):160–7. doi: 10.1177/15269248211003563 (PMC8182333; doi:10.1177/15269248211003563)
Supplement: Supplemental Material, sj-docx-1-pit-10.1177_15269248211003563 - What Are the Burden, Causes, and Costs of Early Hospital Readmissions After Kidney Transplantation? [file sj-docx-1-pit-10.1177_15269248211003563.docx]

**Supplementary Table 2a:** Summary of 30-Day Readmissions with Infection as Reason for Readmission

| **Types of infection** | **Cases**  **N (%)** |
| --- | --- |
| Urinary tract infection | 22 (45.8) |
| Abdominal abscess/infections | 5 (10.4) |
| Access-related infections | 1 (2.1) |
| Surgical site infections | 3 (6.3) |
| Skin infections | 1 (2.1) |
| Others | 16 (33.3) |
| Total | 48 |

**Supplementary Table 2b:** Summary of 30-Day Readmissions with Rejection as Reason for Readmission

| **Types of rejection** | **Cases**  **N (%)** |
| --- | --- |
| Acute tubular necrosis | 1 (2.9) |
| T-cell mediated rejection | 20 (58.8) |
| Antibody mediated rejection | 11 (32.4) |
| Others | 2 (5.9) |
| Total | 34 |
