# Supplementary material for: What Are the Burden, Causes, and Costs of Early Hospital Readmissions After Kidney Transplantation?
Source: Prog Transplant. 2021 Mar 24;31(2):160–7. doi: 10.1177/15269248211003563 (PMC8182333; doi:10.1177/15269248211003563)
Supplement: Supplemental Material, sj-docx-2-pit-10.1177_15269248211003563 - What Are the Burden, Causes, and Costs of Early Hospital Readmissions After Kidney Transplantation? [file sj-docx-2-pit-10.1177_15269248211003563.docx]

**Supplementary Table 3a:** Summary of 90-Day Readmissions with Infection as Reason for Readmission

| **Types of infection** | **Cases**  **N (%)** |
| --- | --- |
| Urinary tract infection | 53 (48.2) |
| Abdominal abscess/infections | 22 (20.0) |
| Pneumonia | 8 (7.3) |
| Access-related infections | 2 (1.8) |
| Surgical site infections | 4 (3.6) |
| Skin infections | 1 (0.9) |
| Others | 20 (18.2) |
| Total | 110 |

**Supplementary Table 3b:** Summary of 90-Day Readmissions with Rejection as Reason for Readmission

| **Types of rejection** | **Cases**  **N (%)** |
| --- | --- |
| Acute tubular necrosis | 2 (4.0) |
| T-cell mediated rejection | 29 (58.0) |
| Antibody mediated rejection | 16 (32.0) |
| Others | 3 (6.0) |
| Total | 50 |
