# Supplementary material for: What Are the Burden, Causes, and Costs of Early Hospital Readmissions After Kidney Transplantation?
Source: Prog Transplant. 2021 Mar 24;31(2):160–7. doi: 10.1177/15269248211003563 (PMC8182333; doi:10.1177/15269248211003563)
Supplement: Supplemental Material, sj-docx-3-pit-10.1177_15269248211003563 - What Are the Burden, Causes, and Costs of Early Hospital Readmissions After Kidney Transplantation? [file sj-docx-3-pit-10.1177_15269248211003563.docx]

**Supplementary Table 4.** Association of Risk Factors with Early Hospital Readmission by Univariable Cox Proportional Hazards Model

| **Variables** | **Early hospital readmission**  **within 30-days** | | **Early hospital readmission within 90-days** | |
| --- | --- | --- | --- | --- |
|  | **HR (95% CI)** | ***P* value** | **HR (95% CI)** | ***P* value** |
| **Recipient age at transplant**, years | 1.00 (0.99, 1.01) | 0.86 | 1.00 (0.99, 1.01) | 0.61 |
| **Recipient sex** Male  Female | *ref* 0.95 (0.72, 1.25) | 0.70 | *ref* 0.97 (0.77, 1.23) | 0.82 |
| **Recipient race** White  Non-White | *ref* 0.98 (0.73, 1.30) | 0.88 | *ref* 1.04 (0.82, 1.32) | 0.70 |
| **Recipient history of diabetes mellitus** | 1.21 (0.90, 1.63) | 0.21 | 1.27 (0.99, 1.64) | 0.06 |
| **Recipient history of chronic lung disease** | 1.86 (1.20, 2.86) | 0.01 | 1.82 (1.24, 2.65) | 0.002 |
| **Recipient history of cardiovascular disease** | 0.96 (0.70, 1.32) | 0.81 | 1.10 (0.85, 1.42) | 0.48 |
| **Recipient body mass index at discharge**, kg/m2 | 1.02 (1.00, 1.05) | 0.06 | 1.02 (1.00, 1.04) | 0.05 |
| **Recipient eGFR at baseline**, ml/min | 1.00 (1.00, 1.01) | 0.60 | 1.00 (0.99, 1.00) | 0.53 |
| **Peak PRA,** % |  |  |  |  |
| 0% | *ref* |  | *ref* |  |
| >0% | 0.92 (0.70, 1.21) | 0.55 | 0.90 (0.71, 1.13) | 0.35 |
| **Time on dialysis**, years | 1.03 (0.99, 1.08) | 0.10 | 1.04 (1.01, 1.08) | 0.02 |
| **Dialysis modality at the time of transplant** |  |  |  |  |
| Conventional hemodialysis | *ref* |  | *ref* |  |
| Home hemodialysis | 1.28 (0.82, 1.99) | 0.27 | 1.20 (0.82, 1.75) | 0.35 |
| Peritoneal dialysis | 1.03 (0.73, 1.45) | 0.88 | 0.97 (0.72, 1.30) | 0.83 |
| Pre-emptive | 1.25 (0.83, 1.87) | 0.29 | 1.08 (0.75, 1.55) | 0.67 |
| **Donor age at transplant,** years | 1.00(1.00, 1.01) | 0.34 | 1.01 (1.00, 1.01) | 0.16 |
| **Type of donation** |  |  |  |  |
| Deceased | *ref* |  | *ref* |  |
| Living | 0.93 (0.71, 1.22) | 0.60 | 0.87 (0.69, 1.09) | 0.22 |
| **Expanded criteria donor (ECD)** |  |  |  |  |
| Non-ECD | *ref* |  | *ref* |  |
| ECD | 1.08 (0.75, 1.55) | 0.68 | 1.14 (0.84, 1.55) | 0.39 |
| **Donation after circulatory death** | 1.16 (0.75, 1.81) | 0.50 | 1.17 (0.80, 1.71 | 0.42 |
| **Length of transplant hospitalization**, days |  |  |  |  |
| ≤7 days | *ref* |  | *ref* |  |
| 8-10 days | 1.05 (0.74, 1.49) | 0.79 | 1.24 (0.92, 1.68) | 0.16 |
| >11 days | 1.35 (0.98, 1.88) | 0.07 | 1.59 (1.19, 2.11) | 0.001 |
| **Delayed graft function** | 1.09 (0.78, 1.52) | 0.60 | 1.25 (0.95, 1.65) | 0.10 |
| **Biopsy-proven acute rejection** | 0.99 (0.51, 1.92) | 0.97 | 1.14 (0.67, 1.95) | 0.64 |
| **Induction therapy at transplant date**  Depleting agent  Non-depleting agent | *ref* 0.66 (0.47, 0.93) | 0.02 | *ref* 0.77 (0.58, 1.02) | 0.07 |
| **Types of calcineurin inhibitor at discharge** Tacrolimus  Cyclosporine | *ref* 1.00 (0.68, 1.47) | 1.00 | *ref* 1.02 (0.74, 1.42) | 0.90 |
| **Transplant era**  2004 - 2007  2008 - 2010  2011 - 2012 | *ref* 1.31 (0.94, 1.82) 1.51 (1.06, 2.13) | 0.11 0.02 | *ref* 1.31 (0.99, 1.72) 1.32 (0.98, 1.78) | 0.06 0.07 |

eGFR, estimated glomerular filtration rate; PRA, panel reactive antibody
