# Supplementary material for: What Are the Burden, Causes, and Costs of Early Hospital Readmissions After Kidney Transplantation?
Source: Prog Transplant. 2021 Mar 24;31(2):160–7. doi: 10.1177/15269248211003563 (PMC8182333; doi:10.1177/15269248211003563)
Supplement: Supplemental Material, sj-docx-4-pit-10.1177_15269248211003563 - What Are the Burden, Causes, and Costs of Early Hospital Readmissions After Kidney Transplantation? [file sj-docx-4-pit-10.1177_15269248211003563.docx]

**Supplementary Table 5:** Reasons for Readmission by Pre-emptive Transplantation
