# Supplementary material for: What Are the Burden, Causes, and Costs of Early Hospital Readmissions After Kidney Transplantation?
Source: Prog Transplant. 2021 Mar 24;31(2):160–7. doi: 10.1177/15269248211003563 (PMC8182333; doi:10.1177/15269248211003563)
Supplement: Supplemental Material, sj-docx-5-pit-10.1177_15269248211003563 - What Are the Burden, Causes, and Costs of Early Hospital Readmissions After Kidney Transplantation? [file sj-docx-5-pit-10.1177_15269248211003563.docx]

**Supplementary Table 1:** Mean and Median Cost of 30-Day Readmission by Hospital Department

| **Department** | **Per early readmission (N_EHRs_=229)** | |
| --- | --- | --- |
|  | **Mean cost (SD) (CAD)** | **Median cost [IQR] (CAD)** |
| Allied health miscellaneous | 79.2 (257.5) | 0 [0, 17.7] |
| Anesthesiology | 59.8 (277.1) | 0 [0, 0] |
| Blood services | 244 (769.7) | 0 [0, 109.1] |
| Cardiology | 641 (1329.2) | 0 [0, 844.6] |
| Emergency | 324.9 (483.1) | 105.0 [0, 488.2] |
| Food | 349.9 (386.4) | 246.5 [149.5, 405.6] |
| Intensive care unit | 148.2 (1184.6) | 0 [0, 0] |
| Laboratory | 1105.4 (1258.8) | 636.6 [361.3, 1458.4] |
| Medical imaging | 486.7 (683.9) | 363.8 [55.1, 537.0] |
| Miscellaneous | 32.5 (131.3) | 0 [0, 0] |
| Nephrology and dialysis | 193.6 (1182.3) | 0 [0, 0] |
| Non-surgical procedure | 49.7 (166.4) | 0 [0, 0] |
| Pharmacy | 2434.2 (3351.6) | 1315.5 [604.3, 2426.8] |
| Psychosocial | 12.5 (53.4) | 0 [0, 0] |
| Rapid response | 28.7 (133.5) | 0 [0, 0] |
| Rehab and palliative care | 47.1 (269.3) | 0 [0, 0] |
| Room and board | 5231.1 (7396.7) | 3360.7 [1641.7, 5765.4] |
| Surgery | 161.6 (632.6) | 0 [0, 0] |
| Unknown | 7.1 (77.4) | 0 [0, 0] |
| Total | 11 606.2 (15 113.1) | 7023.6 [3860.8, 12 404.2] |

EHRs, early hospital readmissions
